# Validity and reliability of the Chinese version of the Jenkins Sleep Scale among university students in China

**DOI:** 10.7717/peerj.19657

**Published:** 2025-07-08

**Authors:** Mengyuan Zhao, Garry Kuan, Ke Zhou, Yee Cheng Kueh

**Affiliations:** 1 Department of Sports Rehabilitation, School of Medical Humanities, Anhui Medical University, Hefei, China; 2 Exercise and Sports Science Programme, School of Health Sciences, Universiti Sains Malaysia, Kubang Kerian, Kelantan, Malaysia; 3 School of Physical Education, Henan University, Keifeng, China; 4 Biostatistics and Research Methodology Unit, School of Medical Sciences, Universiti Sains Malaysia, Kubang Kerian, Kelantan, Malaysia

**Keywords:** Jenkins sleep scale, Confirmatory factor analysis, Psychometric properties, University students

## Abstract

**Background:**

The Chinese version of the Jenkins Sleep Scale (JSS-C) is widely used; however, it lacks formal validation, and its applicability to Chinese university students has not been examined. This study evaluated the validity and reliability of the JSS-C in this population, with a focus on gender differences.

**Methods:**

A cross-sectional study was conducted with 1,534 Chinese university students (29.6% male, 70.4% female; mean age = 19.83 ± 1.54 years) recruited through convenience sampling. Participants completed the JSS-C, which assesses sleep disturbances across four key domains. Confirmatory factor analysis was used to examine construct validity. Internal consistency (Cronbach’s alpha) and composite reliability (CR) were calculated to assess reliability. Measurement invariance testing across gender groups was conducted to evaluate the scale’s robustness.

**Results:**

Confirmatory factor analysis supported a strong one-factor structure for the JSS-C, with excellent model fit indices (*χ*^2^/df = 1.82, Tucker-Lewis index (TLI) = 0.997, comparative fit index (CFI) = 0.999, standardized root mean square residual (SRMR) = 0.007, root mean square error of approximation (RMSEA) = 0.023), exceeding conventional thresholds. Reliability analysis showed high internal consistency (Cronbach’s alpha = 0.840) and composite reliability (CR = 0.844), indicating strong scale stability. The JSS-C also demonstrated full measurement and structural invariance across genders, confirming its unbiased applicability for both male and female students.

**Conclusion:**

The JSS-C is a brief, valid, and reliable instrument for assessing sleep disturbances among Chinese university students. Its standardized scores support meaningful gender-based comparisons.

## Introduction

The ubiquity of sleep across all living organisms underscores its physiological significance (Siegel, 2008). Sleep plays a pivotal role in bodily recuperation, memory integration and consolidation, and represents an indispensable physiological need for humans (Chandrasekaran, Fernandes & Davis, 2020). Impaired sleep has been linked to a spectrum of bodily dysfunctions, encompassing endocrine, metabolic, higher cortical functions, and neurological disorders (Garbarino et al., 2021; Liew & Aung, 2021; Tramontano et al., 2021). The prevalence of sleep disorders is mounting at an alarming rate (Adams et al., 2017; Ai et al., 2022). Research has highlighted a downward trend in the sleep quality of young individuals in recent years (AlDabal & BaHammam, 2011; Edwards & Loprinzi, 2017). University students, in particular, are susceptible to sleep disturbances due to prolonged use of electronic devices such as mobile phones and computers, particularly at night, which can exacerbate sedentary behavior and trigger a vicious cycle of poor sleep (Jenkins et al., 1988; Monterrosa-Castro et al., 2016). In China, the incidence of sleep disorders among university students ranges from 10% to 40%, with a concerning upward trajectory (Fang et al., 2020).

Given the growing emphasis on sleep health assessment, both objective (*e.g.*, polysomnography, actigraphy) and subjective tools (*e.g.*, self-report questionnaires) are widely used (Ohayon et al., 2004). While numerous sleep-specific instruments exist—such as the Pittsburgh Sleep Quality Index (PSQI), Epworth Sleepiness Scale (ESS), and Insomnia Severity Index (ISI)—their length and complexity may limit feasibility in large-scale studies (Chen, Yin & Fang, 2018). The Jenkins Sleep Scale (JSS) addresses this gap as a concise, four-item tool focusing on core sleep disturbances (difficulty falling/staying asleep, nighttime awakenings, and post-sleep fatigue) with demonstrated predictive validity (Jenkins et al., 1988; Monterrosa-Castro et al., 2016). Its brevity makes it ideal for epidemiological research and populations like university students, where time constraints are critical. Despite its translations into multiple languages (Jahrami et al., 2024), the Chinese version (JSS-C) lacks formal psychometric validation, though it has been used in prior studies (Ding et al., 2017; Liu et al., 2021; Wang et al., 2023). Moreover, its applicability to healthy young adults remains understudied. This study therefore evaluates the JSS-C’s reliability, validity, and gender invariance among Chinese university students, hypothesizing robust psychometric properties based on existing evidence.

## Materials & Methods

### Participants

The investigation was conducted between May and July of 2022, employing a convenience sampling method to recruit subjects. Participants were recruited from multiple universities in Jiangsu Province, China, with a focus on health science programs to ensure sample homogeneity in academic background. This multi-institutional approach enhanced the diversity of the sample while maintaining consistency in the participants’ academic exposure to health-related curricula.

Participant inclusion criteria stipulated that participants must: (1) be university students aged between 18 and 26; (2) have Chinese as their mother tongue; and (3) possess normal comprehension abilities. Out of a total of 1,660 data collected, 35 were rejected for incomplete information, while 91 were deemed to have been completed indiscriminately (answer time was too short, and the commonsense question about where the sun rises was answered incorrectly) and were subsequently excluded. The remaining sample comprised 1,534 university students (1,080 of whom were female, representing 70.40% of the total). As suggested by Hair et al. (2006), the minimum sample size required for confirmatory factor analysis is 500. The sample size utilized in this study was 1,534, which is deemed adequate for confirmatory factor analysis.

### Ethics statement

All procedures for this study adhere to the ethical standards of the Universiti Sains Malaysia’s Human Research Ethics Committee (USM/JEPeM/22040240) as well as the Declaration of Helsinki. The data collection and all personal data were anonymized. The study poses minimal risk to participants, as it does not involve procedures such as medical interventions or invasive diagnostics, nor any actions that could cause psychological, mental, or social harm. Prior to completing the questionnaire, participants were required to review the study information, their rights as participants, and relevant precautions. Informed consent was obtained by having participants click the consent button to access the questionnaire. If consent was not granted, the questionnaire would be closed, preventing access to its content.

### Measures

#### Demographic form

Apart from the substantive inquiries presented within the questionnaire, fundamental demographics of the participants were evaluated, encompassing age, academic level, gender, and field of study.

#### Jenkins Sleep Scale

The JSS was initially developed in 1988 by Jenkins et al. (1988) to provide a succinct and efficient instrument for evaluating the frequency and intensity of various sleep disturbances. This survey encompasses difficulties in initiating sleep, frequent nocturnal awakenings, difficulties in maintaining sleep, and subjective experiences of fatigue and sleepiness despite obtaining a typical night’s rest. For example: how often in the past month did you have trouble falling asleep? Participants rated their responses on a 5-point Likert scale, ranging from 0 (indicating no occurrence) to 5 (indicating an occurrence of 22 to 31 days). Higher scores reflect more severe sleep difficulties. The JSS has been translated into multiple languages and widely implemented in various studies (Jahrami et al., 2024). Furthermore, the internal consistency of different translated versions of the JSS has been evaluated using Cronbach’s alpha, consistently demonstrating good reliability (alpha > 0.7) (Duruöz et al., 2018a; Duruöz et al., 2018b; Villarreal-Zegarra et al., 2022). Since the Chinese version of the JSS employed in this study was pre-existing, no further translation was required (Ding et al., 2018).

### Procedure

The study was executed in an online format with due approvals. Initially, the Sojump, a questionnaire website platform (Zhu et al., 2020), was employed to design and produce the electronic survey link. Subsequently, a poster, containing the research topic’s details, the participants’ rights, and instructions on answering the questionnaire, was created. The posters were displayed in areas with a high footfall, such as libraries, canteens, and dormitory bulletin boards, and the relevant school director gave their authorization for this step. Moreover, the survey link was disseminated to diverse class QQ and WeChat groups to increase the study’s exposure. Finally, data was collected anonymously, ensuring that the participants’ responses were not traceable.

### Statistical analysis

Descriptive statistics and correlational analyses were conducted using SPSS 27.0 (IBM Corp., Armonk, NY, USA). Confirmatory factor analysis was performed on the JSS-C using Mplus 8.3. All models were assessed using the covariance matrix, and due to non-normal distributions, robust maximum likelihood method approach (MLM) was employed for estimation. Model fit was compared using the following indices: Cardinality-to-freedom ratio (*χ*^2^/df), Tucker-Lewis Index (TLI), Comparative Fit Index (CFI), standardized root mean square residual (SRMR), and root mean square error of approximation (RMSEA) (Wu, 2011). To achieve good model fit, the *χ*2/df ratio should be as small as possible, while TLI and CFI values close to or greater than 0.95 indicate good or at least acceptable (>0.90) model fit. RMSEA should be ≤0.08, and SRMR should be ≤0.05 or smaller (Wu, 2011). Once an acceptable model fit was established, the reliability (Cronbach’s alpha) and composite reliability (CR) of the JSS-C were calculated using a threshold value of 0.6 for Cronbach’s alpha (Taber, 2018) and CR (Tseng, Dörnyei & Schmitt, 2006). Convergent validity of the scale was assessed using average variance extracted (AVE), with a recommended cut-off value of 0.5 or above (Fornell & Larcker, 1981).

Next, factorial structure was tested in each subgroup, followed by measurement invariance testing in four steps, including a configural model (no constraints), a metric invariant model (with unstandardized item loadings constrained to be equal across groups), a scalar invariant model (with unstandardized item loadings and unstandardized item intercepts simultaneously constrained to be equal across groups), and a strict invariant model (with unstandardized item loadings, unstandardized item intercepts, and error variances simultaneously constrained to be equal across groups) (Wu, 2011). Based on the hierarchical structure of these nested models with increasing constraints, the models were compared with one another. As the *χ*^2^ statistic is sensitive to sample size, we followed the suggestion of Cheung & Rensvold (2002) and used changes in CFI and TLI to evaluate measurement invariance. If ΔCFI and ΔTLI ≤0.01, then the invariance hypothesis cannot be rejected, indicating good model fit.

Finally, an independent samples *t*-test was conducted to examine gender differences in JSS scores. Cohen’s d values proposed by Cohen in 1988 were used to calculate effect sizes. An effect size is considered trivial if it is <0.2, small if it is between 0.2 and 0.5, medium if it is between 0.5 and 0.8, and large if it is >0.8 (Portney & Watkins, 2000).

## Results

The study comprised a total of 1,534 participants, with men constituting a smaller proportion of the sample (29.60%), whereas women constituted the majority (70.40%), the average age is 19.83 years (SD = 1.54). In terms of the participants’ fields of study, a substantial proportion of the sample (85.46%) belonged to the domain of medicine, while the remainder of the participants (14.54%) belonged to other disciplines. With regard to the distribution of participants across different academic years, the freshmen and sophomore classes constituted the major chunk of the sample, accounting for 46.81% and 38.01% respectively, whereas the rest of the academic cohorts comprised a paltry fraction (15.18%) (Table 1).

**Table 1:** Demographic and academic characteristics of the study participants (*N* = 1,534).

Characteristic	Category	Percentage (%)	Number (*n*)
Gender	Male	29.60	454
	Female	70.40	1,080
Field of study	Medicine	85.46	1,311
	Other disciplines	14.54	223
Academic year	Freshmen	46.81	718
	Sophomore	38.01	583
	Other Years	15.18	233
Total participants		100.00	1,534

### Measurement model for the JSS

The JSS-C measurement model, as hypothesized, was unidimensional and comprised of four items. In the initial model fit, the data demonstrated a high level of fit (*χ*^2^ = 3.640, DF = 2, *χ*^2^/*df* = 1.82, RMSEA = 0.023 90% CI [0.000–0.061], CFI = 0.999, TLI =0.997, SRMR = 0.007), which was near perfect. No additional covariance between the item error terms was required to improve model fit, thus confirming the suitability of the initial model as the final model. The factor loadings for each item were between 0.704 and 0.811, exceeding the recommended value of 0.5 (Hair et al., 2010). Table 2 presents the standardized factor loads and average scores for each item measured by the JSS-C. Multivariate skewness and kurtosis were found to be significant (*p* < 0.05, based on both univariate and multivariate tests), indicating a non-normal distribution. Additionally, all items met the standard cut-off point for corrected item-total correlations of above 0.50 (Hair et al., 2010).

**Table 2:** Item characteristics of the JSS-C.

Items	Factor loadings	Mean	SD	Skewness	Kurtosis	r_it_
S1	0.772	1.14	1.21	1.123	0.842	0.693
S2	0.742	0.89	1.082	1.407	1.913	0.663
S3	0.811	1.16	1.345	1.218	0.810	0.717
S4	0.704	1.34	1.325	0.943	0.281	0.638
Total-JSS		4.52	4.095	1.080	0.981	

**Notes.**

SD standard deviation r_it_ Corrected item-total correlation

Furthermore, internal consistency analyses revealed that the Cronbach’s alpha value was 0.840, and the CR value was 0.844, both exceeding the recommended value of 0.6 (Taber, 2018; Tseng, Dörnyei & Schmitt, 2006). These results demonstrate the satisfactory reliability of the JSS-C structure. The AVE value calculated for the scale as a whole was 0.575, indicating good convergent validity.

### Measurement invariance across genders

Table 3 presents the model fit indices *χ*^2^/df, CFI, TLI, and RMSEA, which all surpass the standard values, demonstrating the excellent fit of the models to the data. When comparing the metric invariance model to the configural invariance model, the scalar invariance model to the metric invariance model, and the strict invariance model to the scalar invariance model, the differences in ΔCFI and ΔTLI were all below 0.01. Consequently, based on the small values of ΔCFI (−0.001) and ΔTLI (0.000), we can assume strict invariance of this model across both men and women.

**Table 3:** Measurement invariance testing results of the JSS-C across genders (*N* = 1,534).

Model	*χ* ^2^	df	*χ*^2^/df	P	CFI	TLI	RMSEA (90%CI)	ΔCFI	ΔTLI
Male	0.709	2	0.355	0.7015	1.000	1.007	0.000 (0.000–0.068)	–	–
Female	3.885	2	1.943	0.1433	0.998	0.995	0.030 (0.000–0.074)	–	–
Configural	4.177	4	1.044	0.3826	1.000	1.000	0.008 (0.000–0.056)	–	–
Metric	5.187	7	0.741	0.6372	1.000	1.002	0.000 (0.000–0.037)	0.000	0.002
Scalar	10.809	10	1.081	0.3726	1.000	0.999	0.010 (0.000–0.041)	0.000	−0.003
Strict	16.383	14	1.170	0.2905	0.999	0.999	0.015 (0.000–0.039)	−0.001	0.000

**Notes.**

*χ*^2^ chi-square goodness of fit df degrees of freedom CFI Compartative Fit Index TLI Tucker–Lewis index RMSEA root mean square error of approximation 90% CI 90% confidence intervals ΔCFI CFI difference Δ TLI TLI difference

### Gender differences in JSS-C

The present study employed independent sample *t*-tests to compare the differences in scores of participants between genders on each entry and overall scores of the JSS-C. The results are presented in Table 4. Interestingly, female participants exhibited higher overall scores on sleep disturbances than male participants. Although there were numerical differences in scores between genders on each JSS-C entry, none of them reached statistical significance. Furthermore, all Cohen’s d values indicated trivial effect sizes, suggesting that gender differences in JSS-C scores were minuscule. The Bonferroni correction test results also indicated that there were no statistically significant differences across all items.

**Table 4:** Gender comparison of JSS-C scores in the university student cohort (M± SD).

Items	Male (*n*= 454)	Female (*n*= 1,080)	Total sample (*N* = 1,534)	*t*	—Cohen’s d —
S1	1.15 ± 1.31	1.14 ± 1.17	1.14 ± 1.21	0.148	0.008
S2	0.86 ± 1.11	0.90 ± 1.07	0.89 ± 1.08	−0.62	0.037
S3	1.19 ± 1.43	1.14 ± 1.31	1.16 ± 1.35	0.68	0.036
S4	1.26 ± 1.34	1.37 ± 1.32	1.34 ± 1.33	−1.52	0.083
Total-Score	4.46 ± 4.36	4.55 ± 3.98	4.52 ± 4.10	−0.36	0.022

**Notes.**

*t* *t*-value of independent sample *t*-test —Cohen’s d— absolute value of Cohen’s d

## Discussion

This study evaluated the psychometric properties of the JSS-C using confirmatory factor analysis, demonstrating strong validity and reliability. The one-factor model exhibited an excellent fit to the data (*χ*^2^/*df* = 1.82, RMSEA = 0.023, CFI = 0.999, TLI = 0.997, SRMR = 0.007), with all four items loading adequately onto the latent construct. Internal consistency was high (Cronbach’s alpha = 0.840), and both composite reliability (CR > 0.6) and average variance extracted (AVE > 0.5) met established thresholds, confirming the scale’s robust construct validity. These results align with prior validations of the JSS in other languages (Duruöz et al., 2018a; Duruöz et al., 2018b; Duruöz et al., 2019; Unal-Ulutatar & Ozsoy-Unubol, 2020; Pallarés-Sanmartín et al., 2019). Importantly, the JSS-C demonstrated full measurement invariance across genders, supporting its unbiased applicability for assessing sleep disturbances in Chinese university students.

The present investigation conducted the first-ever JSS-C screening using the measurement invariance test in a Chinese sample, which is applicable to both male and female university students. The assessment of nested models was accomplished through the comparison of ΔCFI and ΔTLI, with values less than 0.01, signifying the non-rejection of the equivalence hypothesis and the fitting of the nested models. The multiple confirmatory factor analysis findings revealed that the data supported the configural invariance, weak invariance, strong invariance, and strict invariance of the JSS-C. The outcomes suggested that JSS-4 was strictly invariant across genders. Thus, the scale can be used to compare differences in sleep disorders among university students of different genders. Any differences in results are attributable to the characteristics of the population rather than the scale itself.

Although the results of this study indicated that there was no significant difference in sleep problems between male and female university students, this did not necessarily mean that sleep problems did not differ between genders. In fact, previous research has shown that there are some differences in sleep quality between men and women (Krystal, Prather & Ashbrook, 2019; Pengo, Won & Bourjeily, 2018). For example, women are typically more prone to insomnia and difficulty waking up than men (Krystal, Prather & Ashbrook, 2019). Additionally, women experience changes in their sleep quality during physiological stages such as menstrual cycles, pregnancy, and menopause (Pengo, Won & Bourjeily, 2018). Therefore, to fully investigate gender differences in sleep quality at different stages, it is necessary to consider multiple factors, including physiological and psychological factors.

While not statistically significant, the total JSS score for women was higher than that for men. Therefore, the results of this study are consistent with Tibubos et al.’s (2020) research on sleep disorders in specific gender groups, suggesting that women are more likely to experience sleep problems to some extent. It is reasonable to observe that sleep disorders do not differ between genders in the population studied here. This is because previous research has shown that sleep problems increase with age and are positively correlated with psychological stress, which is more prominent in women (Kalmbach et al., 2018). The majority of participants in this study were freshmen and sophomores, who are relatively young and may have fewer physiological and psychological differences compared to older university students (Li et al., 2020). Therefore, the difference in sleep quality between men and women in this age group may not be significant. If the study were expanded to include individuals of different ages and occupations, different results may emerge.

There are limitations to the current study that cannot be ignored. Firstly, the sample predominantly consisted of medical students (85% of participants), which may limit the generalizability of the findings to students from other academic disciplines. Future studies should include more diverse student populations to validate the scale’s applicability across different fields of study. Secondly, the research design employed for this study was cross-sectional, and self-reported data collection was used, which may have introduced bias into the understanding of the participants. Moreover, the study had limited indicators for testing the reliability of the JSS-C. Future research could further use additional indicators to verify the psychometric properties of the scale. Additionally, future studies could collect data from the same cohort of subjects at multiple time points to analyse the invariance of the scale over time. Although 1,534 data were collected and included in the analysis, a more comprehensive analysis with a larger sample size from different regions would have produced more generalizable results. Lastly, other aspects of the Chinese version of the Jenkins Sleep Scale could be studied. For example, the reliability and validity of the scale in other group populations could be investigated, or the relationship between the scores on the scale and other relevant factors could be explored. These studies would help to deepen our understanding of the manifestation and influencing factors of sleep problems in the Chinese population and provide more information and support for research in related fields.

## Conclusions

In summary, the JSS-C has exhibited strong reliability, validity and measurement invariance with regards to gender within the university population, thus rendering it a viable assessment tool for sleep-related issues. The attainment of cross-gender invariance in this study bolsters the utility of the JSS-C, as it furnishes empirical substantiation for utilizing the scale in gauging gender-specific sleep disturbances.

##  Supplemental Information

10.7717/peerj.19657/supp-1 Supplemental Information 1 Raw data

10.7717/peerj.19657/supp-2 Supplemental Information 2 Codebook for converting data

## Additional Information and Declarations

### Data Availability

The following information was supplied regarding data availability: The raw measurements are available in the Supplementary File.
