# Supplementary material for: Validity and reliability of the Chinese version of the Jenkins Sleep Scale among university students in China
Source: PeerJ. 2025 Jul 8;13:e19657. doi: 10.7717/peerj.19657 (PMC12248221; doi:10.7717/peerj.19657)
Supplement: Supplemental Information 2 [file peerj-13-19657-s002.docx]

**Note for the raw data:**In the item of gender: “1” means male, “2” means female.

S1 represents the first item "Have trouble falling asleep?", S2 represents "Wake up several times per night?", S3 represents "Have trouble staying asleep (including waking far too early)?", S4 represents "Wake up after your usual amount of sleep feeling tired and worn out?", and Ssleep represents the total score for sleep disturbances. "0" = Not at all, "1" = 1-3 days, "2" = 4-7 days, "3" = 8-14 days, "4" = 15-21 days, "5" = 22-31 days.
